# Zearalenone Promotes Hepatic Stellate Cell Activation and Early Profibrotic Tendency in the Liver

**DOI:** 10.3390/biom16050644

**Published:** 2026-04-26

**Authors:** Lige Bao, Yongze Huang, Jiaxin Bao, Yitong Lu, Chunli Chen, Zhiyong Wu, Jichang Li

**Affiliations:** 1 College of Veterinary Medicine, Northeast Agricultural University, Harbin 150030, China; baolige@neau.edu.cn (L.B.); huangyongze2023@163.com (Y.H.); z11634@neau.edu.cn (J.B.); luyitong126@126.com (Y.L.); chunli.chen@neau.edu.cn (C.C.); wuzhiyong@neau.edu.cn (Z.W.); 2 Heilongjiang Key Laboratory for Animal Disease Control and Pharmaceutical Development, Northeast Agricultural University, Harbin 150030, China

**Keywords:** zearalenone, hepatic stellate cell, HSC activation, liver injury

## Abstract

Zearalenone (ZEA) is a mycotoxin widely present in cereals, feeds, and foods, posing a persistent threat to human and animal health. Hepatic fibrosis is a pathological process characterized by excessive extracellular matrix (ECM) deposition. Chronic liver injury caused by sustained oxidative stress can initiate the development of early hepatic fibrosis. However, whether liver injury induced by ZEA can trigger hepatic stellate cell (HSC) activation and promote early profibrotic responses remains unclear. The aim of this study was to assess whether ZEA-induced liver injury promotes HSC activation and early profibrotic responses. To address this, we established a BALB/c mouse exposure model and used the murine HSC line (JS-1) for in vitro validation. The results showed that ZEA exposure caused structural damage in hepatic tissue and produced an incomplete bridging pattern of collagen thickening suggestive of an early profibrotic tendency. ZEA shaped a proinflammatory microenvironment by activating the IκBα/NF-κB axis and induced the TGF-β1/Smad2/3 pathway, accompanied by Smad7 suppression, thereby promoting HSC activation and the expression of fibrosis-related genes. ZEA also altered autophagy-related markers in liver tissue and JS-1 cells. Pharmacological inhibition with chloroquine partially attenuated ZEA-induced upregulation of α-SMA and collagen I/III, suggesting that autophagy-related processes may be involved in ZEA-associated HSC activation and early ECM deposition. In summary, ZEA promotes HSC activation and early profibrotic changes in the liver and is associated with inflammatory activation, TGF-β1/Smad signaling, and altered autophagy-related activity. These findings provide a basis for further investigation into the mechanisms underlying ZEA-induced early profibrotic remodeling in the liver.

## 1. Introduction

Mycotoxins are toxic products of fungal secondary metabolism, and contamination by them has become a global problem affecting the animal-feed and human-food chains [1]. This contamination occurs at every stage, from the harvest of raw grains, storage, transportation, and processing through to final feeding or consumption [2,3]. Contamination of grains poses a potential risk to livestock systems that rely on grain-based feed [4], where it can reduce production efficiency and cause economic losses [5]. Zearalenone (ZEA) is among the most frequently detected mycotoxins worldwide [6], and recent years’ monitoring indicates persistently high levels [7,8]. Its biosynthesis is regulated by multiple factors, including humidity, temperature, environmental pH, and precipitation [9,10].

ZEA contamination is mainly observed in temperate and relatively humid cereal-producing regions, with a distribution across Europe, the Americas, temperate Asia, and parts of Africa [11]. In Asia (e.g., China and South Korea), the detection rates in feed and cornmeal are as high as 96.9% and 88%, respectively [12,13]. Widespread detection is also reported in Europe and the Americas: 80% in U.S. corn by-products, 77.5% in Brazilian cattle feed, 85% in German corn, 82% in Finnish wheat, 93.3% in Croatian pig feed, and 85% in Romanian animal feed [14,15,16,17,18]. Multiple global surveys further indicate that many African countries (Kenya, Ethiopia, Tanzania, Madagascar, Côte d’Ivoire, and Nigeria) bear a higher burden of ZEA contamination [8]. Beyond its widespread detection in grains and feeds, ZEA is also frequently detected in human biological samples. In Japanese adults, urinary detection rates reached 90% in the Tokai region and 61% in Hokkaido [19]. Among agricultural workers in Nigeria’s Nasarawa and Niger States, the ZEA detection rate was 58% [20]. In Portugal, approximately 76% of urine samples from lactating mothers and children tested positive for ZEA, and 55.26% of breast milk samples were contaminated as well [21].

As the primary site of first-pass metabolism and detoxification, the liver is among the major target tissues for ZEA toxicity [22,23]. It has been demonstrated that ZEA exposure in mice causes pathological changes, including destruction of hepatic lobular architecture and inflammatory cell infiltration in the liver [24,25]. Our previous work demonstrated that ZEA induces significant liver injury in BALB/c mice, as evidenced by increased ALT, AST, and ALP [26]. Concurrently, ZEA suppressed antioxidant defenses by downregulating the activities of CAT and SOD and reducing GSH and T-AOC levels. It also increased ROS production and inhibited the Nrf2/Keap1-mediated antioxidant pathway, thereby impairing ROS-scavenging capacity and disrupting hepatic redox homeostasis [26].

Notably, persistent ROS accumulation and redox imbalance are not only hallmark events of liver injury but can also act as upstream drivers that amplify inflammatory and profibrotic responses [27]. Accumulating evidence indicates that chronic oxidative stress can stimulate hepatic immune cells to produce profibrogenic mediators and can directly activate hepatic stellate cells (HSC), thereby promoting the initiation and progression of liver fibrosis [28,29]. Liver fibrosis is essentially a reversible wound-healing response, whose core feature is the excessive deposition of extracellular matrix (ECM) after acute or persistent injury [30]. Within a microenvironment of sustained oxidative stress and inflammation, hepatocellular injury becomes difficult to resolve, leading to persistent ECM accumulation and the gradual formation of fibrotic scar tissue [30,31]. Multiple studies suggest that HSC activation is pivotal in the progression of hepatic fibrosis [32,33,34,35]. During activation, HSC upregulate α-smooth muscle actin (α-SMA) and elevate the expression of other intracellular microfilaments [31]. The activated HSC then migrates toward sites of injury and secretes large amounts of ECM, especially type I (COL1A1, Collagen I) and type III (COL3A1, Collagen III) collagen, ultimately forming fibrotic scars [30,31,36].

In recent years, mycotoxin exposure has also been reported to activate and drive hepatic fibrosis. The aflatoxin B1 (AFB1) causes persistent hepatocellular injury and promotes the secretion of proinflammatory cytokines, thereby activating HSC and leading to hepatic fibrosis [37]. The fumonisin B1 exposure upregulates transforming growth factor-β1 (TGF-β1), thereby inducing HSC activation through the TGF-β1/α-SMA pathway and resulting in collagen deposition [38]. In addition to oxidative stress, inflammation, and the TGF-β/Smad axis, autophagy also exerts a critical regulatory function in hepatic fibrosis. Many studies demonstrate that autophagy enhances activation of HSC [32,33,39,40,41,42]. Autophagy stimulates the loss of lipid droplets (LDs) in HSC, providing energy for activation and promoting HSC survival [33,42]. It has been found that various autophagy inhibitors can markedly attenuate HSC activation [32,41,42].

At present, the molecular mechanisms by which ZEA induces liver injury are not fully defined. Our previous work showed that ZEA can cause hepatic injury by inducing oxidative stress. However, whether this injury further drives quiescent HSC activation and contributes to an early profibrotic tendency has not been systematically demonstrated. Therefore, we established a mouse model of ZEA exposure and complemented it with experiments in the murine hepatic stellate cell line JS-1 to assess whether ZEA-mediated liver injury is associated with HSC activation and early profibrotic responses.

## 2. Materials and Methods

### 2.1. Chemicals and Reagents

Zearalenone (ZEA) was purchased from Prebolab (Qingdao, China). Chloroquine (CQ) was purchased from MedChemExpress (Monmouth Junction, NJ, USA). RPMI-1640 medium was purchased from Thermo Fisher Scientific (Waltham, MA, USA), antibiotic-antimycotic solution was purchased from Solarbio (Beijing, China), and fetal bovine serum (FBS) was purchased from Biological Industries (Kibbutz Beit Haemek, Israel). TRIzol™ reagent was purchased from Invitrogen (Carlsbad, CA, USA). The PrimeScript™ RT reagent kit was obtained from Takara (Tokyo, Japan), and the SYBR Green reagent was purchased from Selleck (Houston, TX, USA). RIPA lysis buffer, phenylmethanesulfonyl fluoride (PMSF), and phosphatase inhibitor were purchased from Beyotime (Shanghai, China). The Cell Counting Kit-8 (CCK-8) and enhanced chemiluminescence (ECL) reagent were obtained from Meilun Biotechnology (Dalian, China). Polyvinylidene difluoride (PVDF) membranes were purchased from Merck Millipore (Billerica, MA, USA). The primary and secondary antibodies used in this study, together with their sources and dilution ratios, are listed in Supplementary Table S5. Unless otherwise specified, all other chemicals and reagents used in this study were of analytical grade.

### 2.2. Experimental Animals and Dosing Regimen

All SPF-grade BALB/c mice (male, 5-week-old) used in this study were purchased from Liaoning Changsheng Biotechnology Co., Ltd. (Benxi, China), animal License No. SCXK (Liao) 2020-0001. The animal study protocol was approved by the Animal Welfare and Ethics Committee of the College of Veterinary Medicine, Northeast Agricultural University (protocol code NEAUEC202303143; date of approval: 20 October 2023). After a 7-day acclimation period in the animal facility, 75 BALB/c mice were randomized to 5 groups: blank control (Con), vehicle control (Vcon), low-dose ZEA (20 mg/kg, ZEA20), medium-dose ZEA (40 mg/kg, ZEA40), and high-dose ZEA (80 mg/kg, ZEA80), with specific grouping and dosing regimens detailed in Supplementary Table S1. The Con group received no gavage treatment and was maintained under the same housing conditions as the other groups. The Vcon group received 0.1 mL of corn oil by oral gavage daily. ZEA solutions at the indicated doses were prepared in corn oil and administered by oral gavage (0.1 mL/mouse/day) for 30 consecutive days. On day 31, mice in all groups were anesthetized and then euthanized for necropsy. Liver tissues were harvested and weighed, a portion was stored at −80 °C, and another was fixed in 4% paraformaldehyde (PFA) or 2.5% glutaraldehyde. The animal exposure regimen used in the present study was the same as that described in our previous report [26,43]. Biochemical indicators of liver injury, including ALT, AST, and ALP, from this same animal cohort were published previously [26]. The present study established a subchronic high-dose ZEA toxicology model in mice, and the rationale for the in vivo dose selection is provided in Supplementary Material Section S1.

### 2.3. Cell Culture and Treatments

The mouse hepatic stellate cell line (JS-1) was purchased from FengHui Biological Co., Ltd. (Changsha, China). JS-1 was routinely cultured in complete RPMI-1640 with 1% antibiotic-antimycotic and 10% FBS at 37 °C under 5% CO_2_. When JS-1 cells reached the exponential growth phase, the supernatant was removed, and the cells were gently washed. ZEA was dissolved in DMSO and diluted in culture medium so that the final concentration of DMSO was 0.1% in all groups. The JS-1 cells were then treated with RPMI-1640 medium containing ZEA at final concentrations of 10, 20, or 30 μM for 12 h. After treatment, cells and culture supernatants were harvested for subsequent assays. Cells were divided into 4 groups: control (Con), low-dose ZEA (ZEA10), medium-dose ZEA (ZEA20), and high-dose ZEA (ZEA30). Control group cells received the vehicle only. Detailed grouping information is provided in Supplementary Table S2.

To investigate the role of autophagy in ZEA-induced liver fibrosis, the autophagy inhibitor chloroquine (CQ) was included. CQ was dissolved in DMSO and diluted in culture medium so that the final concentration of DMSO was 0.1%. Four groups were established: control group (Con), ZEA 20 group (20 μM, 12 h), CQ group (10 μM), and ZEA 20 + CQ group (CQ 10 μM + ZEA 20 μM, co-treated 12 h). After the above treatments, cells and culture supernatants were collected for subsequent assays. Detailed grouping information is provided in Supplementary Table S3.

### 2.4. JS-1 Cell Viability Assay

JS-1 cells (5 × 10^4^ cells/well) were seeded in 96-well plates and allowed to adhere for 12 h, then exposed to ZEA (0, 5, 10, 20, 30, 40, 60, 80 μM) for a further 12 h. Cell viability was quantified using a CCK-8 assay according to the manufacturer’s instructions. Briefly, the culture medium was replaced with fresh medium containing CCK-8 reagent, and plates were incubated at 37 °C for 30 min. Absorbance at 450 nm was recorded with a microplate reader (Thermo Fisher Scientific, Waltham, MA, USA), and viability was calculated using the kit formula.

### 2.5. H&E and Masson’s Staining of the Mouse Liver

Fresh liver samples were gently rinsed three times with physiological saline and then fixed in 4% PFA. After fixation, the liver tissues were routinely dehydrated, cleared, paraffin-embedded, and sectioned at 4 μm. Finally, after deparaffinization and rehydration, the samples were subjected to H&E and Masson’s staining following the instructions. Following staining and subsequent processing, the prepared sections were examined and recorded using a light microscope (Leica, Wetzlar, Germany). Representative H&E and Masson images were obtained from liver sections from three mice per group, and histological assessment was performed in a blinded manner.

### 2.6. Preparation and Observation of Liver TEM Samples

Fresh mouse liver tissue was cut into approximately 1 mm^3^ and fixed at 4 °C. After fixation, the liver tissues were routinely processed by PBS washing, osmium tetroxide post-fixation, graded dehydration, and embedding. Ultrathin liver sections (55–70 nm) were prepared using an ultramicrotome. Following staining with uranyl acetate and lead citrate, the tissues were examined and imaged under a transmission electron microscope (Hitachi HT7650, Tokyo, Japan) to assess ultrastructural changes in hepatic cells. Representative TEM images were obtained from liver samples from three mice per group.

### 2.7. RNA Preparation and Real-Time qPCR Analysis

The mouse liver tissues (100 mg) were weighed and placed in pre-chilled 1.5 mL tubes (RNase-free), and 1 mL of TRIzol reagent was added. The samples were fragmented with a pre-chilled tissue homogenizer (Jingxin, Shanghai, China) three times for 1 min each. For JS-1 cells, 1 mL of TRIzol was added to each well, and JS-1 cells were gently scraped, and the lysates were collected into RNase-free tubes. The total RNA was then isolated from the samples using the standard TRIzol protocol. After extraction, RNA pellets were resuspended in 30–50 μL of DEPC-treated water and gently pipetted repeatedly to fully dissolve the precipitate. Total RNA concentration and purity for each sample were assessed using the NanoDrop 2000 spectrophotometer (Thermo Fisher Scientific, Waltham, MA, USA). To ensure consistency in subsequent experiments, RNA from all samples was diluted or adjusted to the same working concentration using DEPC-treated water. Subsequently, genomic DNA removal and reverse transcription were performed according to the PrimeScript RT reagent kit instructions. RT-qPCR was performed using the SYBR Green method on a LightCycler 96 system (Roche, Basel, Switzerland). Reaction mixtures were prepared according to the manufacturer’s instructions. Supplementary Table S4 summarizes the primer sequences for each gene. Ct values for target and reference genes were obtained from the system, and *GAPDH* was used as the internal reference. ΔCt was calculated as (Ct_target-Ct_GAPDH). The control group was used as calibration to obtain ΔΔCt, and relative mRNA expression was expressed as 2^−ΔΔCt^.

### 2.8. Western Blotting

The total protein from liver tissue and JS-1 cells was isolated using pre-chilled RIPA lysis buffer. The working lysis buffer was prepared by adding 10 μL of PMSF and 20 μL of phosphatase inhibitor to each 1 mL of RIPA buffer. Briefly, liver tissue (50 mg) was rapidly excised, weighed, and placed in a pre-chilled EP tube, followed by the addition of 500 μL of pre-chilled working lysis buffer. The tissue was thoroughly homogenized and then lysed on ice for 30 min (three rounds of homogenization, 1 min each). For JS-1 cells, 300–500 μL of working lysis buffer was added to each well, and the cell lysates were collected and further lysed on ice for 30 min. After lysis, the samples were centrifuged at 12,000× *g* for 15 min at 4 °C, and the supernatants were collected. Total protein concentrations were then determined using a BCA protein assay kit at 562 nm. All samples were adjusted to the same working concentration, mixed with 5 × SDS-PAGE loading buffer, and heat-denatured in a metal bath.

Western blotting was performed according to standard procedures. A total of 30 μg of protein was loaded per lane, and proteins were separated by SDS-PAGE at a constant voltage of 80 V for 30 min, followed by 120 V for 70 min. After electrophoresis, the separating gel was cut according to the expected molecular weights of the target proteins, and the proteins were transferred onto PVDF membranes using the sandwich transfer method. Protein transfer was carried out at a constant current of 200 mA in an ice bath, and the transfer time was adjusted according to the molecular weight of the target proteins. After transfer, the PVDF membranes were washed with TBST and blocked with rapid blocking solution for 20 min at room temperature (RT). The primary antibodies were diluted according to the manufacturer’s recommended ratios and incubated with the membranes overnight at 4 °C. After incubation, the membranes were thoroughly washed with TBST and then incubated with the corresponding secondary antibodies for 90 min at RT. Protein bands were visualized using an enhanced chemiluminescence kit, and images were acquired with a Tanon imaging system (Tanon, Shanghai, China). Target protein intensities were quantified by gray-value analysis using ImageJ software (version 1.53k). The sources and dilution ratios of all antibodies are listed in Supplementary Table S5.

### 2.9. JS-1 Cells Immunofluorescence Staining

JS-1 cells (1 × 10^6^/well) were seeded in 12-well plates. When confluence reached 50–60%, the medium was discarded and washed with DPBS, and treatments were applied as in Section 2.2. Afterward, JS-1 cells were washed with PBS and fixed in 1 mL 4% PFA for 20 min at RT. Fixed cells were washed in PBS, permeabilized with Triton X-100 (20 min, RT), and washed again. Blocking was performed with 1% BSA (30 min, RT), followed by incubation with primary antibodies (overnight, 4 °C). After completion, species-matched fluorophore-conjugated secondary antibodies were applied in the dark (60 min, RT). Nuclei were counterstained with DAPI after a final PBS rinse, and images were captured on a fluorescence microscope. Fluorescence intensity was quantified in three random fields per group from three independent experiments using ImageJ software. Antibody sources and dilutions are listed in Supplementary Table S5.

### 2.10. Statistical Analysis

Data are expressed as mean ± SD from at least three independent experiments (n ≥ 3). Analyses and graphing were performed in GraphPad Prism 9.0; additional analyses used SPSS 25.0. Two-group comparisons used unpaired Student’s *t*-tests; multiple-group comparisons used one-way ANOVA, subsequently Tukey’s post-hoc test. The *p* value < 0.05 was considered significant. In figures, * *p* < 0.05; ** *p* < 0.01; ns, not significant. For in vivo data, asterisks indicate differences vs. Vcon; for in vitro data, vs. Con. In CQ-inhibition assays, asterisks denote differences between the two groups connected by a line.

## 3. Results

### 3.1. ZEA Exposure Induces Hepatic Injury in Mice

**Figure 1:**
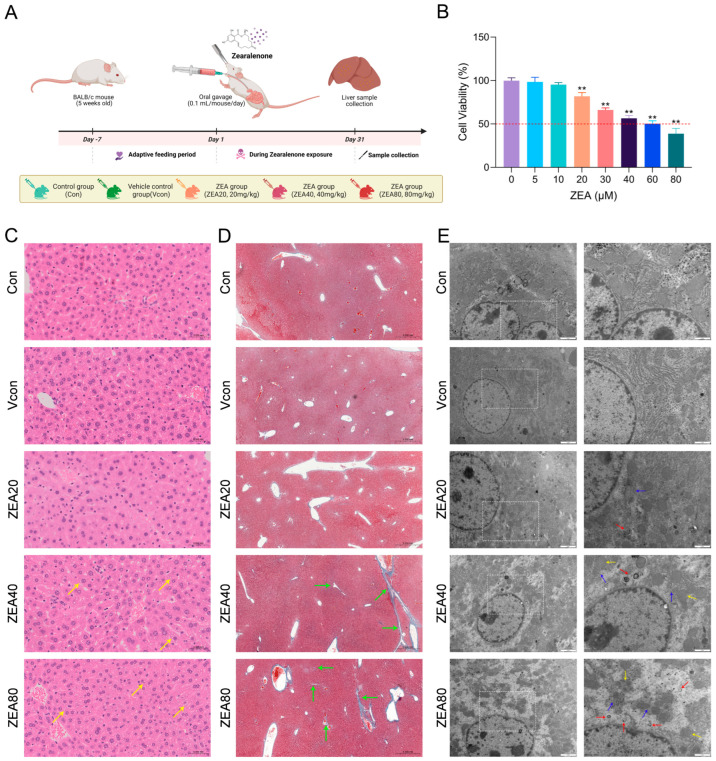
ZEA induces structural injury in mouse liver tissue. (**A**) Experimental timeline and group allocation for the mouse ZEA exposure model. (**B**) CCK-8 assessment of JS-1 cell viability after ZEA exposure. (**C**) H&E staining of mouse liver (400×; 0.05 mm); yellow arrows indicate vacuolar degeneration of hepatocytes. (**D**) Masson’s staining of mouse liver (50×; 0.5 mm); green arrows indicate blue-stained collagen fibers. (**E**) TEM images of hepatic ultrastructure (10,000×; 2 μm and 20,000×; 1 μm; Acquisition parameters: Magnification: 10,000×; High Voltage: 100 kV); The TEM images on the right are higher-magnification views of the white dashed-box regions indicated on the left. Yellow arrows indicate mitochondrial swelling and cristae disruption, blue arrows indicate endoplasmic reticulum damage, and red arrows indicate autophagosomes. Representative H&E, Masson, and TEM images are shown from three mice per group; histological assessment was performed in a blinded manner. The statistical significance, represented by ** for *p* < 0.01, were observed in comparisons between each group and the ZEA (0 μM) group.

To determine whether ZEA exposure is accompanied by histopathological and ultrastructural alterations associated with early profibrotic changes, a mouse model of ZEA exposure was established (Figure 1A). BALB/c mice were euthanized following 30 days of exposure to ZEA (20, 40, or 80 mg/kg). We then assessed whether ZEA exposure induced histopathological and ultrastructural liver damage as well as early profibrotic alterations. To assess ZEA-induced liver damage, H&E staining was used to evaluate histopathological changes. As shown in Figure 1C, the Con and Vcon groups exhibited intact hepatic lobular architecture with normal cell morphology. In contrast, the ZEA40/80 groups showed prominent perinuclear vacuolar degeneration of hepatocytes, with denser vacuolization in the ZEA80 group. Masson staining results are presented in Figure 1D. Compared with the Con and Vcon groups, the ZEA40 group displayed thickened blue-stained collagen fibers in the portal regions, as well as around blood vessels. For the ZEA80, portal expansion was more pronounced, and slender collagen fibers extended into adjacent hepatic lobules, indicating a tendency toward incomplete bridging. Ultrastructural changes are shown in Figure 1E. Hepatocytes in the Con and Vcon groups exhibited orderly structures and regular mitochondrial morphology. By contrast, the ZEA40/80 groups exhibited mitochondrial swelling with disrupted cristae and dilation of the rough endoplasmic reticulum. Autophagosomes were observed at all ZEA doses and were notably increased at the ZEA80 dose. Together, the findings support that ZEA causes structural and ultrastructural damage in mouse hepatic tissue and suggest an early profibrotic tendency in the liver.

### 3.2. ZEA Induces Profibrotic Marker Changes in Liver Tissue and JS-1 Cells

**Figure 2:**
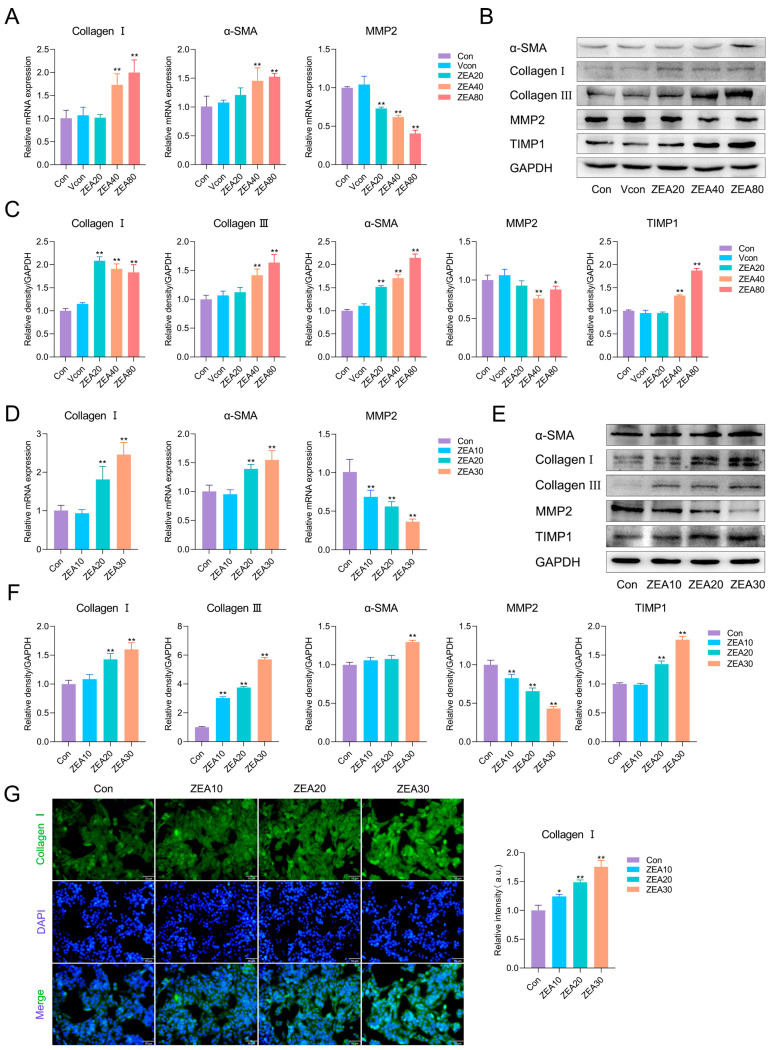
ZEA induces early profibrotic changes in liver tissue and JS-1 cells. (**A**) RT-qPCR analysis of *α-SMA*, *Collagen I*, and *MMP2* mRNA levels in mouse liver (n = 3). (**B**) Representative Western blot bands for liver tissue. (**C**) Western blot quantification of α-SMA, Collagen I, Collagen III, MMP2, and TIMP1 in mouse liver (n = 3). (**D**) RT-qPCR analysis of *α-SMA*, *Collagen I*, and *MMP2* mRNA levels in JS-1 cells (n = 4). (**E**) Representative Western blot bands for JS-1 cells. (**F**) Western blot quantification of α-SMA, Collagen I, Collagen III, MMP2, and TIMP1 in JS-1 cells (n = 3). (**G**) Immunofluorescence staining of Collagen I in JS-1 cells after ZEA exposure (10 μm). Statistical significance was denoted as * *p* < 0.05 and ** *p* < 0.01. Unless otherwise specified, comparisons were made relative to the Vcon group for in vivo experiments and to the Con group for in vitro experiments. Asterisks above connecting lines indicate the significance of comparisons between the connected groups. To avoid layout redundancy, this explanatory note is provided only in Figure 2 and applies to Figure 2, Figure 3, Figure 4, Figure 5 and Figure 6. The original Western blot images can be found in the Supplementary Materials.

To determine whether ZEA induces early profibrotic alterations in vivo, we assessed α-SMA together with major ECM-related markers, including Collagen I, Collagen III, matrix metalloproteinase 2 (MMP2), and its inhibitor tissue inhibitor of metalloproteinases 1 (TIMP1), in liver tissue. As depicted in Figure 2A, relative to the Vcon group, ZEA exposure markedly upregulated the mRNA levels of *α-SMA* and *Collagen I* and downregulated *MMP2* mRNA levels. As indicated by Figure 2B,C, TIMP1, α-SMA, Collagen I, and Collagen III protein levels were markedly upregulated in the ZEA-exposed groups relative to Vcon, while MMP2 protein was markedly reduced.

Because HSC activation is the critical initiating point in fibrosis development, the mouse JS-1 hepatic stellate cell line was used for in vitro experiments. We first used a CCK-8 assay to determine the effect of ZEA on JS-1 cell viability (Figure 1B). Following exposure to ZEA (5–80 μM), a significant change in viability was observed from 20 μM onward relative to the Con group; therefore, we selected 10, 20, and 30 μM exposures (12 h) for subsequent assays. As illustrated in Figure 2D, ZEA dramatically increased *α-SMA* and *Collagen I* mRNA levels and dramatically decreased *MMP2* mRNA levels in JS-1 cells. As shown in Figure 2E,F, TIMP1, α-SMA, Collagen I, and Collagen III protein levels were significantly upregulated in the ZEA groups, while MMP2 was dramatically downregulated. Furthermore, immunofluorescence staining for Collagen I (Figure 2G) revealed markedly enhanced fluorescence intensity in all ZEA dose groups relative to the Con group. Collectively, these findings show that ZEA promotes the secretion of ECM proteins and suppresses collagen-degrading capacity, thereby exhibiting an early profibrotic tendency.

### 3.3. ZEA Activates NF-κB Signaling and Drives Inflammatory Responses

**Figure 3:**
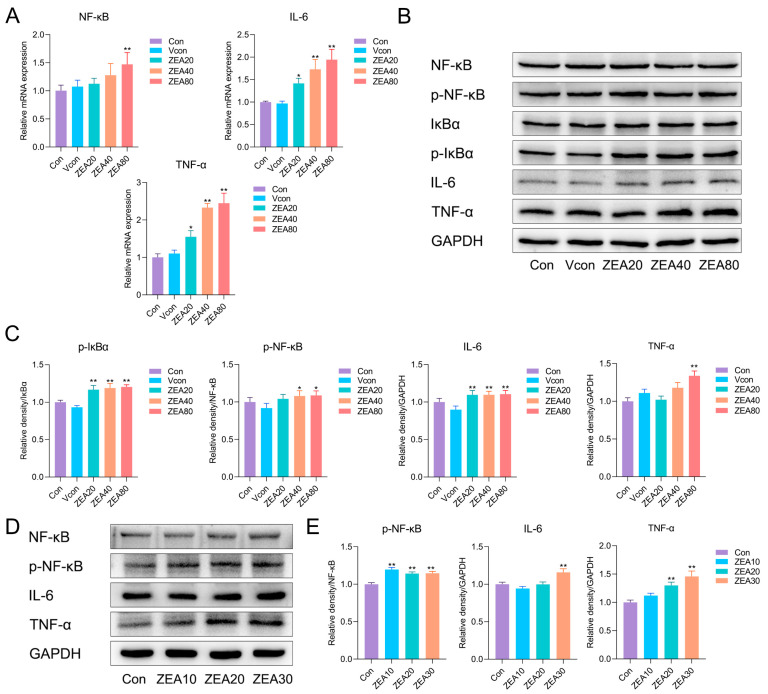
ZEA triggers canonical NF-κB and promotes expression of proinflammatory mediators. (**A**) RT-qPCR analysis of *TNF-α*, *IL-6*, and *NF-κB* mRNA levels in mouse liver (n = 3). (**B**) Representative Western blot bands for mouse liver. (**C**) Western blot quantification of p-IκBα, p-NF-κB, TNF-α, and IL-6 in mouse liver (n = 3). (**D**) Representative Western blot bands for JS-1 cells. (**E**) Western blot quantification of p-NF-κB, IL-6, and TNF-α in JS-1 cells (n = 3). Statistical significance was denoted as * *p* < 0.05 and ** *p* < 0.01. The original Western blot images can be found in the Supplementary Materials.

Studies across diverse etiologies and experimental models of hepatic fibrosis have shown that inflammation triggered by chronic hepatocellular injury is a key pathogenic mechanism driving fibrogenesis [30,31]. Persistent inflammation typically precedes and promotes fibrotic progression, and inflammatory cytokines play central roles in this process [30,31]. In this study, we further examined hepatic levels of NF-κB, IκBα, and the proinflammatory cytokines TNF-α and IL-6. As shown in Figure 3A, RT-qPCR revealed that ZEA exposure dramatically increased the *NF-κB*, *TNF-α*, and *IL-6* mRNA levels in liver tissue. Consistent with these findings, Western blotting demonstrated significant upregulation of p-NF-κB, p-IκBα, TNF-α, and IL-6 protein levels in ZEA-exposed groups relative to Vcon (Figure 3B,C). In JS-1 cells, ZEA also significantly increased p-NF-κB, TNF-α, and IL-6 protein levels (Figure 3D,E). These results indicate that ZEA activates the NF-κB pathway and elicits inflammatory responses, thereby providing an inflammatory basis for early profibrotic remodeling.

### 3.4. ZEA Activates the TGF-β1/Smad2/3 Axis to Drive Profibrotic Signaling

**Figure 4:**
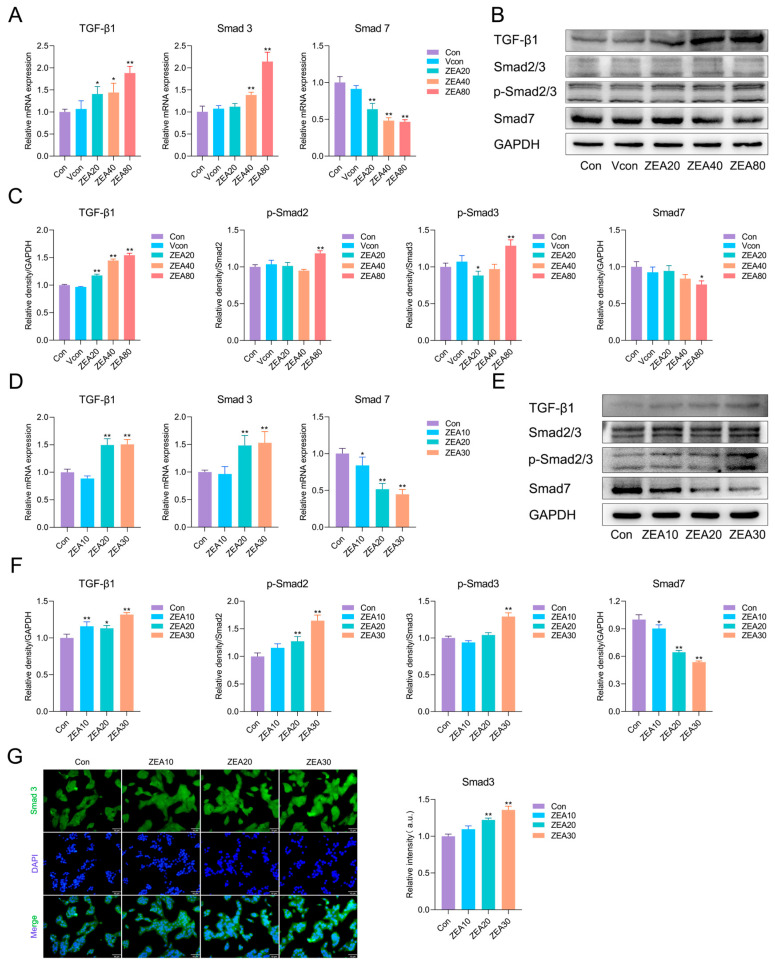
ZEA activates the TGF-β1/Smad2/3 axis to drive profibrotic responses. (**A**) RT-qPCR analysis of *TGF-β1*, *Smad3*, and *Smad7* mRNA levels in mouse liver (n = 3). (**B**) Representative Western blot bands for liver tissue. (**C**) Western blot quantification of fibrosis-associated proteins in mouse liver (n = 3). (**D**) RT-qPCR analysis of *TGF-β1*, *Smad3*, and *Smad7* mRNA levels in JS-1 cells (n = 4). (**E**) Representative Western blot bands for JS-1 cells. (**F**) Western blot quantification of fibrosis-associated proteins in JS-1 cells (n = 3). (**G**) Immunofluorescence staining of Smad3 in JS-1 cells after ZEA exposure (10 μm). For Smad2/3 and p-Smad2/3, the upper and lower bands detected within the same lane were assigned according to the expected molecular weights and quantified separately to derive the p-Smad2/Smad2 and p-Smad3/Smad3 ratios. Statistical significance was denoted as * *p* < 0.05 and ** *p* < 0.01. The original Western blot images can be found in the Supplementary Materials.

Beyond inflammatory mediators, TGF-β1 is widely recognized as a principal profibrotic cytokine and induces HSC activation in a manner dependent on Smad2/3 [31,44]. Therefore, we measured mRNA and protein levels of key components within the TGF-β/Smad signaling cascade in mouse liver tissue and JS-1 cells. As presented in Figure 4A, ZEA exposure dramatically upregulated hepatic *TGF-β1* and *Smad3* mRNA levels and downregulated *Smad7* mRNA. At the protein level (Figure 4B,C), compared with the Vcon group, ZEA increased TGF-β1 expression and the ratios of p-Smad2/Smad2 and p-Smad3/Smad3, whereas Smad7 expression levels were decreased. In JS-1 cells (Figure 4D), the mRNA changes were consistent with the in vivo results, with *TGF-β1* and *Smad3* upregulated, whereas *Smad7* was downregulated. As shown in Figure 4E,F, ZEA exposure significantly elevated TGF-β1 expression and the ratios of p-Smad2/Smad2 and p-Smad3/Smad3, and significantly reduced Smad7 protein expression levels relative to the Con group. Further immunofluorescence staining for Smad3 (Figure 4G) revealed a marked increase in fluorescence intensity after ZEA exposure. Taken together, ZEA activates the TGF-β1/Smad2/3 axis both in vivo and in vitro, suggesting that it enhances HSC activation and profibrotic transcriptional programs, thereby promoting fibrotic progression.

### 3.5. ZEA Alters Autophagy-Related Markers in Hepatic Tissue and JS-1 Cells

**Figure 5:**
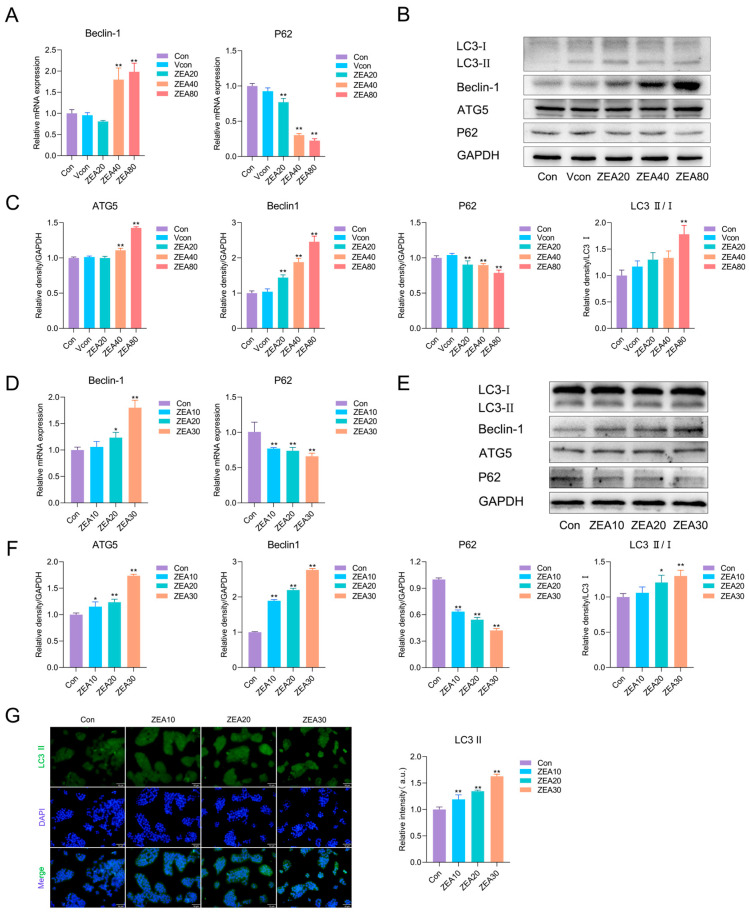
ZEA alters autophagy-related markers in mouse liver tissue and JS-1 cells. (**A**) RT-qPCR analysis of *Beclin-1* and *P62* mRNA levels in mouse liver (n = 3). (**B**) Representative Western blot bands for liver tissue. (**C**) Western blot quantification of Beclin-1, ATG5, P62, and LC3-II/I in mouse liver (n = 3). (**D**) RT-qPCR analysis of *Beclin-1* and *P62* levels in JS-1 cells (n = 4). (**E**) Representative Western blot bands for JS-1 cells. (**F**) Western blot quantification of Beclin-1, ATG5, P62, and LC3-II/I in JS-1 cells (n = 3). (**G**) Immunofluorescence staining of LC3-II in JS-1 cells after ZEA exposure (10 μm). Statistical significance was denoted as * *p* < 0.05 and ** *p* < 0.01. The original Western blot images can be found in the Supplementary Materials.

Growing evidence indicates that, in addition to inflammation and the TGF-β1/Smad axis, autophagy-related processes may be associated with early profibrotic remodeling in the liver. Therefore, we measured autophagy-related markers to explore their association with ZEA-induced early profibrotic responses. In liver tissue (Figure 5A), ZEA exposure significantly downregulated *P62* mRNA levels and upregulated *Beclin-1* mRNA levels relative to the Vcon. At the protein level (Figure 5B,C), ZEA exposure significantly raised the LC3-II/LC3-I ratio and the ATG5 and Beclin-1 expression levels, whereas P62 protein levels declined. In JS-1 cells (Figure 5D), analysis of gene expression revealed that ZEA exposure markedly upregulated *Beclin-1* mRNA levels and downregulated *P62* mRNA levels. Consistently, relative to the Con group, ZEA significantly increased the LC3-II/LC3-I ratio and the protein levels of Beclin-1 and ATG5 and significantly reduced P62 protein levels (Figure 5E,F). Further LC3-II immunofluorescence (Figure 5G) showed a markedly enhanced fluorescence intensity after ZEA exposure. These findings indicate that ZEA markedly alters the levels of autophagy-related markers in both hepatic tissue and JS-1 cells.

### 3.6. Chloroquine Partially Attenuates ZEA-Induced Early Profibrotic Responses in JS-1 Cells

**Figure 6:**
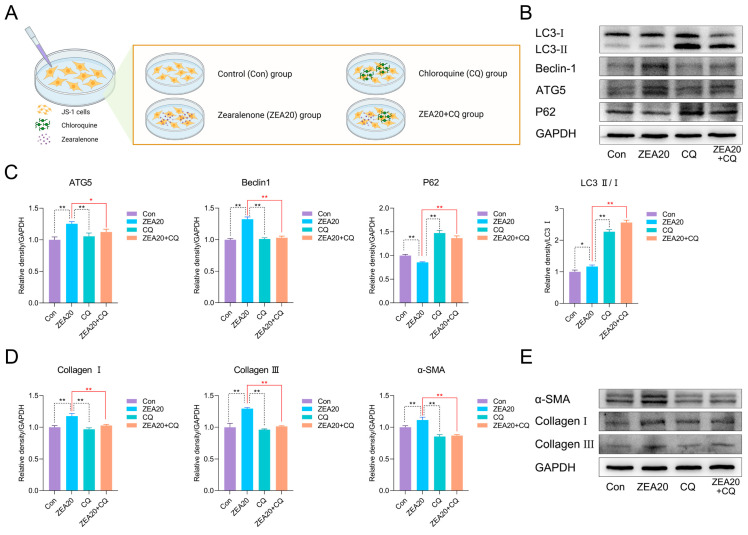
Chloroquine partially attenuates ZEA-associated profibrotic marker changes in JS-1 cells. (**A**) Schematic of group design for CQ inhibition in JS-1 cells. (**B**) Representative Western blot bands of autophagy markers in JS-1 cells. (**C**) Western blot quantification of Beclin-1, ATG5, P62, and LC3-II/I in JS-1 cells under CQ inhibition (n = 3). (**D**) Western blot quantification of α-SMA, Collagen I, and Collagen III in JS-1 cells under CQ inhibition (n = 3). (**E**) Representative Western blot bands of fibrosis-related markers in JS-1 cells. Statistical significance was denoted as * *p* < 0.05 and ** *p* < 0.01. The original Western blot images can be found in the Supplementary Materials.

To further assess whether autophagy-related processes may contribute to ZEA-induced early profibrotic responses, JS-1 cells were incubated with CQ, an autophagy inhibitor, and autophagy-and fibrosis-related markers were measured; the experimental groups are shown in Figure 6A. First, we examined autophagy-related proteins after CQ and/or ZEA treatment. As shown in Figure 6B,C, compared with the ZEA20 group, CQ + ZEA20 co-treatment significantly downregulated ATG5 and Beclin-1 protein levels and markedly elevated the LC3-II/LC3-I ratio and P62 levels. The concomitant accumulation of LC3-II and P62 is consistent with late-stage inhibition of autophagy, suggesting that CQ interfered with autophagy-related processing in this model.

In JS-1 cells, we next evaluated protein levels of fibrogenic activation markers (Collagen I, Collagen III, α-SMA). As illustrated in Figure 6D,E, relative to the ZEA20 group, the ZEA20 + CQ group exhibited dramatically decreased Collagen I, Collagen III, and α-SMA protein levels. Taken together, CQ partially mitigated ZEA-induced early profibrotic responses, suggesting that a CQ-sensitive autophagy-related process may be involved in ZEA-associated early profibrotic tendency.

## 4. Discussion

Zearalenone (ZEA) ranks among the most frequently detected mycotoxins worldwide and can damage multiple tissues, including the liver, intestine, and reproductive organs [45]. It is a ubiquitous cereal-associated mycotoxin and, owing to its heat stability, may persist through food/feed processing, resulting in widespread dietary exposure [21,45]. Our previous work demonstrated that ZEA causes significant hepatic redox imbalance and oxidative stress injury [26]. Persistent oxidative stress is increasingly recognized as an upstream determinant that amplifies inflammatory and profibrogenic programs during chronic liver injury, thereby promoting a tendency toward hepatic fibrosis [31], and the initiating step is the activation of HSC [30,31]. Therefore, we used BALB/c mice and JS-1 cells to evaluate whether ZEA exposure induces an early profibrotic tendency and to explore whether autophagy-related processes may be involved in ZEA-associated early profibrotic responses.

Building on this redox phenotype, we next assessed whether ZEA exposure is accompanied by histopathological and ultrastructural hepatic injury and early extracellular matrix remodeling. Histopathology revealed vacuolar degeneration of hepatocytes in the ZEA groups. Consistently, transmission electron microscopy showed marked hepatocellular damage. Masson staining in the ZEA80 group revealed thickened collagen in portal areas and around blood vessels, with slender collagen fibers extending toward adjacent hepatic lobules, indicating a tendency toward incomplete bridging. Taken together, ZEA not only disrupts hepatic tissue architecture but also promotes early collagen fiber deposition, providing morphological support for an early profibrotic tendency in the liver.

Hepatic fibrosis is a reversible tissue-repair reaction, defined by extracellular matrix (ECM) deposition in response to liver injury [30,31]. During chronic liver injury, ECM synthesis exceeds its rate of degradation, and consequently leads to hepatic fibrosis [30]. ECM deposition is closely linked to activation of HSC [46]. Under physiological conditions, HSC are quiescent (qHSC) and contain vitamin A-rich lipid droplets (LDs) [31]. Upon liver injury, qHSC become activated, lose their lipid-storing phenotype, strongly upregulate α-SMA [47], proliferate, and differentiate into myofibroblasts (activated HSC, aHSC) [47], which are a major source of ECM proteins [48]. The fibrotic ECM is predominantly composed of Collagen I and Collagen III [48]. ECM homeostasis is tightly regulated by matrix metalloproteinases (MMPs) and their endogenous inhibitors, tissue inhibitors of metalloproteinases (TIMPs) [30,49]. MMPs are key enzymes that degrade hepatic ECM, acting on both collagenous and non-collagenous substrates [30]. As endogenous inhibitors of MMPs, TIMPs antagonize MMP activity, thereby enhancing ECM deposition and advancing hepatic fibrogenesis [32].

Lin et al. showed that combined exposure to deoxynivalenol (DON), ZEA, and AFB1 dramatically upregulated hepatic α-SMA protein levels in mice, and Masson staining indicated increased collagen deposition [50]. Several mycotoxins have also been shown to induce a fibrotic tendency in the liver [37,38,51]. For example, AFB1 induces hepatic fibrosis in mice by activating HSC and increasing the TGF-β1, Collagen I, Collagen III, and α-SMA expression levels [52]. Ochratoxin A (OTA) increases the degree of fibrosis around the portal tracts in the mouse liver [53]. Consistent with these reports, our research found that ZEA exposure significantly enhanced the HSC activation marker α-SMA levels in liver tissue. It also upregulated the expression of the major collagen fiber components, Collagen I and Collagen III. Concomitantly, ZEA downregulated MMP2 and upregulated TIMP1. In JS-1 cells, these related proteins exhibited consistent expression changes following ZEA exposure. Taken together, ZEA promotes ECM protein secretion and, by limiting collagen degradation, jointly drives early ECM deposition and a profibrotic tendency.

HSC activation is regulated by multiple factors, among which proinflammatory cytokines and TGF-β are pivotal drivers. Persistent inflammation often precedes fibrosis [30], and ROS, LPS, and several proinflammatory mediators (IL-6, TNF-α, IL-1β, and leptin) promote HSC activation [31,54]. Activated HSC act as both sources and targets of inflammatory mediators, forming a hub that links inflammation and fibrosis [55]. They continue to secrete cytokines such as IL-6, IL-1β, and TGF-β, thereby further promoting HSC activation and hepatic fibrogenesis [56]. NF-κB is a central transcriptional hub in this process, and redox imbalance and ROS can act as upstream modulators that prime or amplify NF-κB-dependent transcription, thereby promoting the expression of proinflammatory cytokines and other profibrotic genes, enhancing HSC survival, and amplifying inflammatory injury [30,55,57]. Previous studies reported that ZEA activates classical NF-κB signaling in mouse liver, accompanied by increased inflammatory cytokines [25,58,59]. In line with these findings, we observed significant increases in the phosphorylation of NF-κB and IκBα in both liver tissue and in JS-1 cells after ZEA exposure, together with upregulation of IL-6 and TNF-α. These findings support the involvement of the IκBα/NF-κB axis in ZEA-induced inflammatory activation, thereby promoting proinflammatory cytokine expression and contributing to a hepatic inflammatory milieu that favors HSC activation.

In chronic liver injury, redox imbalance may facilitate TGF-β1 activation and downstream Smad signaling, thereby contributing to a profibrogenic microenvironment [28,29]. TGF-β is the principal profibrotic growth factor driving HSC activation and collagen synthesis [60]. Among its three major isoforms, TGF-β1 is the principal isoform associated with hepatic fibrosis [30]. Once activated, TGF-β1 binds its receptor complex and specifically initiates Smad signaling, which enhances transcription of target genes, including pro-collagen I and pro-collagen III [30]. Mechanistically, binding of TGF-β1 to its receptors (TGF-βR1) leads to receptor dimerization and recruitment of Smad2 and Smad3 proteins. These proteins are then phosphorylated, and phosphorylated Smad2/3 associate with Smad4 to form complexes [30,61]. The complexes enter the nucleus and control the expression of genes linked to fibrosis [30,61]. TGF-β1 can further disrupt ECM homeostasis by suppressing MMPs and inducing TIMPs [62]. In contrast, Smad7 acts as an inhibitory regulator of this pathway by preventing Smad2/3 activation and facilitating degradation of the TGF-β receptor complex [63]. Previous studies have shown that ZEA can activate TGF-β1 signaling in other tissues and cell models [64,65]. In agreement with prior findings, we observed that ZEA exposure significantly upregulated TGF-β1 expression, enhanced Smad2/3 phosphorylation, and suppressed the antifibrotic regulator Smad7, indicating that the TGF-β1/Smad axis was continuously activated. Therefore, we infer that ZEA induces a redox-disturbed and inflammatory hepatic microenvironment, accompanied by activation of the TGF-β1/Smad signaling pathway. Together with the observed increases in α-SMA and Collagen I/III expression, these findings support the notion that ZEA may promote early ECM deposition.

Autophagy is a cellular self-digestive process that degrades and metabolizes cytoplasmic components by delivering cargo to lysosomes via autophagosomes [39]. It is dynamically controlled by multiple proteins that act at distinct stages of the pathway [39]. Autophagy-related markers such as LC3, P62, Beclin-1, and ATG5 are commonly used to evaluate autophagy-associated activity, and the combined assessment of LC3-II and P62 protein levels has become a classic marker combination for monitoring autophagy activity and flux [32,39,66]. Previous studies have shown that ZEA exposure increases the expression of Beclin-1, ATG5, and LC3-II while reducing P62 in mouse liver and hepatocyte models [67,68,69]. Consistent with these findings, in our study, ZEA significantly upregulated ATG5 and Beclin-1 in the liver, elevated the LC3-II/LC3-I ratio, and decreased P62. In vitro, JS-1 cells exposed to ZEA exhibited changes in autophagy marker mRNA and proteins that were concordant with those in vivo. Together, these findings indicate that ZEA alters autophagy-related markers in vivo and in vitro and suggest the involvement of autophagy-related processes.

Autophagy is now considered one of the key processes in hepatic fibrogenesis [39]. Thoen et al. reported that HSC activation is accompanied by an increase in autophagy [39]. Inhibition of autophagy with BafA1 suppresses the transition of qHSC to a myofibroblast-like phenotype (aHSC), and other autophagy inhibitors, including 3-MA, CQ, and HCQ, likewise attenuate HSC activation [39]. In the quiescent state, HSC are rich in vitamin A-containing LDs [32,40]. Autophagy degrades LDs and thereby provides energy for HSC activation [33]. Multiple studies consistently show that autophagy promotes HSC activation, while pharmacologic inhibition of autophagy or knockout of key autophagy genes effectively mitigates the fibrotic tendency [32,33,40,41]. To further assess whether autophagy-related processes may contribute to ZEA-associated early profibrotic responses, JS-1 cells were co-treated with ZEA and the autophagy inhibitor CQ. CQ markedly altered the ZEA-induced autophagy-related profile, as evidenced by downregulation of ATG5 and Beclin-1 together with accumulation of LC3-II and P62, indicating interference with late-stage autophagy-related processing. Importantly, CQ co-treatment significantly reduced the protein levels of α-SMA, Collagen I, and Collagen III compared with ZEA treatment alone. These findings show that CQ can partially alleviate these changes in JS-1 cells, suggesting that a CQ-sensitive autophagy-related process may be involved in ZEA-associated early profibrotic responses.

In addition, the present study employed a non-lethal subchronic high-dose exposure model, and the doses used were substantially higher than the typical environmental or dietary exposure levels encountered by humans or livestock. Therefore, the current findings should be interpreted mainly as mechanistic evidence, and caution is needed when extending them to real-world chronic low-dose exposure conditions. In addition, although JS-1 cells generally retained relatively high viability under ZEA10, ZEA20, and ZEA30 treatment conditions, some of the signaling changes observed under the higher in vitro exposure conditions, particularly at ZEA30, may also partly reflect the influence of cellular stress. At the same time, the current evidence regarding the role of autophagy in this study should still be regarded as exploratory rather than definitive mechanistic evidence. Our data mainly indicate that ZEA exposure can induce early profibrotic changes and suggest that autophagy-related processes may be involved in this response. Nevertheless, this conclusion still has certain limitations. First, chloroquine, as a late-stage autophagy inhibitor, may exert off-target effects. Second, this study did not further validate the role of the autophagy pathway in this process through knockdown of autophagy-related targets. Therefore, although our findings provide preliminary pharmacological support for the possible involvement of autophagy-related processes in ZEA-induced early profibrotic remodeling, the currently available evidence is still insufficient to definitively establish the precise mechanistic role of autophagic flux in this process. The present study only provides a preliminary exploration of the possible role of autophagy-related processes in ZEA-induced early profibrotic remodeling in the liver. Future studies will still be required to investigate this issue in a more systematic, in-depth, and comprehensive manner, so as to further clarify the significance and mechanistic contribution of autophagy-related processes to the early profibrotic tendency induced by ZEA.

## 5. Conclusions

**Figure 7:**
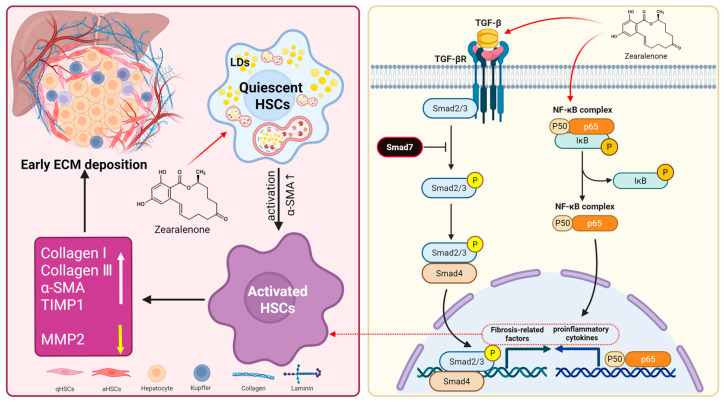
ZEA activates HSCs through multiple pathways and induces an early profibrotic tendency in the liver.

In summary, our in vivo and in vitro findings indicate that ZEA exposure promotes HSC activation and elicits early signs of ECM deposition. Mechanistically, ZEA shapes a pro-inflammatory milieu by activating the IκBα/NF-κB axis, and it elevates TGF-β1 while enhancing Smad2/3 phosphorylation and lowering Smad7, which together favor the expression of fibrosis-related genes. Notably, ZEA also altered autophagy-related markers in liver tissue and JS-1 cells, and pharmacological inhibition with chloroquine partially attenuated the upregulation of α-SMA and collagen (Figure 7).

Collectively, these findings preliminarily indicate that ZEA can induce early profibrotic changes in the liver and suggest that autophagy-related processes may be involved in this response. However, the precise significance and mechanistic contribution of autophagy and autophagic flux in this process still require further investigation. Overall, the present study lays a foundation for further exploring the mechanisms underlying the early profibrotic tendency in the liver associated with ZEA exposure.

## Data Availability

The original contributions presented in this study are included in the article/Supplementary Materials. Further inquiries can be directed to the corresponding author.

## Supplementary Materials

The following supporting information can be downloaded at: https://www.mdpi.com/article/10.3390/biom16050644/s1, Supplementary Materials and methods, including S1. ZEA dosage selection criteria [70,71,72,73,74,75,76,77,78,79,80]; Table S1: Summary of routes of administration, dosages, and dosing regimens used in animal experiments; Table S2: Experimental grouping of JS-1 cells for ZEA exposure; Table S3: Experimental grouping for CQ intervention assay in JS-1 cells; Table S4: Primer sequences for RT-qPCR analysis; Table S5: Antibody and dilution rate for Western blot/IF experiments. Original images of Figure 2B,E, Figure 3B,D, Figure 4B,E, Figure 5B,E and Figure 6B,E can be found in Supplementary Materials.

## Abbreviations

The following abbreviations are used in this manuscript:

ZEA	Zearalenone
DON	Deoxynivalenol
OTA	Ochratoxin A
AFB1	Aflatoxin B1
CQ	Chloroquine
ECM	Extracellular matrix
HSC	Hepatic stellate cells
aHSC	Activated hepatic stellate cells
qHSC	Quiescent hepatic stellate cells
JS-1	The mouse hepatic stellate cell line
LDs	Lipid droplets
α-SMA	α-smooth muscle actin
Collagen I	Collagen type I
Collagen III	Collagen type III
MMP2	Matrix metalloproteinase 2
TIMP1	Tissue inhibitor of metalloproteinases 1
TGF-β1	Transforming growth factor-β1
TGF-βRI	Transforming growth factor-β1 receptor
Smad2/3/7	SMAD family members 2, 3, and 7
LC3	Microtubule-associated protein 1 light chain 3
ALT	Alanine aminotransferase
AST	Aspartate aminotransferase
ALP	Alkaline phosphatase
CAT	Catalase
SOD	Superoxide dismutase
GSH	Glutathione
T-AOC	Total antioxidant capacity
ROS	Reactive oxygen species
Nrf2	Nuclear factor erythroid 2-related factor 2
Keap1	Kelch-like ECH-associated protein 1
LPS	Lipopolysaccharide
NF-κB	Nuclear factor kappa B
IκBα	Inhibitor of kappa B alpha
IL-6	Interleukin-6
IL-1β	Interleukin-1β
TNF-α	Tumor necrosis factor-α
