# Supplementary material for: Zearalenone Promotes Hepatic Stellate Cell Activation and Early Profibrotic Tendency in the Liver
Source: Biomolecules. 2026 Apr 26;16(5):644. doi: 10.3390/biom16050644 (PMC13204578; doi:10.3390/biom16050644)
Supplement: Supplementary file 1 [file biomolecules-16-00644-s001.zip › biomolecules-4249505 -Supplementary Material.pdf]

Supplementary material

Zearalenone promotes hepatic stellate cell activation and early profibrotic tendency in the liver

Lige Bao [1,2], Yongze Huang [1,2], Jiaxin Bao [1,2], Yitong Lu [1,2], Chunli Chen [1,2], Zhiyong Wu [1,2] and Jichang Li [1,2,*]

1 College of Veterinary Medicine, Northeast Agricultural University, Harbin, 150030, P.R. China
2 Heilongjiang Key Laboratory for Animal Disease Control and Pharmaceutical Development, Northeast Agricultural University, Harbin, 150030, P.R. China
* Correspondence: lijichang@neau.edu.cn

Jichang Li

Author Address: College of Veterinary Medicine, Northeast Agricultural University, Harbin, 150030, P.R. China

E-mail address: lijichang@neau.edu.cn

List of Supplementary Material

Supplementary materials and methods
Supplementary tables
Table S1. Summary of routes of administration, dosages, and dosing regimens used in animal experiments.
Table S2. Experimental grouping of JS-1 cells for ZEA exposure
Table S3. Experimental grouping for CQ intervention assay in JS-1 cells
Table S4. Primer sequences for RT-qPCR analysis.
Table S5. Antibody and dilution rate for Western blot/IF experiments.

Supplementary materials and methods

S1. ZEA dosage selection criteria

Zearalenone (ZEA) is widely detected in cereals, feed, and food, and the contamination levels in some samples can reach relatively high concentrations. According to a report from the European Food Safety Authority (EFSA), the highest concentrations of ZEA contamination reached 823-3000 μg/kg during 2005-2010 [70]. Herrman, Zinedine, and others also reported that ZEA contamination levels could be as high as 600 mg/kg [71]. In addition, ZEA has been detected in corn samples from Nigeria at levels up to 17.5 mg/kg [72], while corn samples from Egypt were reported to contain relatively high levels of ZEA ranging from 9.8-38.4 mg/kg [73]. Furthermore, some studies have reported extremely high levels of ZEA contamination in food samples. For example, ZEA was detected in food samples from the United States at concentrations as high as 2900 mg/kg [71].

The dosing regimen used in the present study was selected within a non-lethal dose range designed to induce significant toxic responses without causing death, thereby establishing a subchronic high-dose mechanistic toxicology model. The purpose of this model was to reliably induce measurable liver injury within a controllable experimental period and to further investigate whether sustained ZEA exposure is sufficient to trigger an early profibrotic tendency and related mechanistic changes. The dose selection was based mainly on previous in vivo toxicological studies and our preliminary experiments. Previous reports have shown that the oral LD_{50} of ZEA in mice is approximately 500 mg/kg [74,75]. After reviewing extensive literature, we found that a dose of 40 mg/kg has been used in multiple subchronic in vivo studies to induce obvious toxic responses without causing significant mortality [76-80]. Based on the above literature evidence and our preliminary experimental results, 40 mg/kg was selected as the reference middle dose, corresponding to approximately 8% of the reported LD_{50}. On this basis, 20, 40, and 80 mg/kg/day were adopted as low-dose, middle-dose, and high-dose groups, respectively, to establish a dose gradient for mechanistic investigation of subchronic toxicity while remaining within the non-lethal range. Therefore, these dose levels were intended only to support mechanistic toxicology research under controlled experimental conditions and should not be interpreted as representing the typical environmental or dietary exposure levels encountered by humans or livestock in daily life.

Table S1. Summary of routes of administration, dosages, and dosing regimens used in animal experiments

Group ID	Number of Animals	Exposure Group	Exposure Dose	Administration Volume	Route of Administration	Frequency of Administration
1	15	Blank control group (Con group)	—	None	None	None
2	15	Vehicle control group (Vcon group)	—	0.1mL	oral gavage	QD × 30 d
3	15	ZEA（ZEA20 group）	20 mg/kg	0.1mL	oral gavage	QD × 30 d
4	15	ZEA（ZEA40 group）	40 mg/kg	0.1mL	oral gavage	QD × 30 d
5	15	ZEA（ZEA80 group）	80 mg/kg	0.1mL	oral gavage	QD × 30 d

Table S2. Experimental grouping of JS-1 cells for ZEA exposure

Group ID	Exposure Group	Vehicle	ZEA	Exposure Duration	Remarks
1	Control group (Con group)	0.1% DMSO	0 μM	12 h	Cells treated with vehicle only
2	ZEA 10 group	0.1% DMSO	10 μM	12 h	Cells exposed to 10 μM ZEA
3	ZEA 20 group	0.1% DMSO	20 μM	12 h	Cells exposed to 20 μM ZEA
4	ZEA 30 group	0.1% DMSO	30 μM	12 h	Cells exposed to 30 μM ZEA

Table S3. Experimental grouping for CQ intervention assay in JS-1 cells

Group ID	Exposure Group	Vehicle	CQ	ZEA	Exposure Duration	Remarks
1	Con group	0.1% DMSO	0 μM	0 μM	12 h	Cells treated with vehicle only
2	ZEA20 group	0.1% DMSO	0 μM	20 μM	12 h	Cells exposed to 20 μM ZEA
3	Chloroquine (CQ) group	0.1% DMSO	10 μM	0 μM	12 h	Cells exposed to 10 μM CQ
4	CQ+ZEA20 group	0.1% DMSO	10 μM	20 μM	12 h	Cells co-treated with 20 μM ZEA and 10 μM CQ

Table S4. Primer sequences for RT-qPCR analysis

Gene name	Forward primer（5′ – 3′）	Reverse primer（5′ – 3′）
GAPDH	AAGATTGTCAGCAATGCATCCTG	AGTTGCTGTTGAAGTCGCAG
Collagen I	CGATGGATTCCCGTTCGAGT	GAGGCCTCGGTGGACATTAG
MMP2	AGACACTGGTCGCAGTGATG	CCATGGTAAACAAGGCTTCATGG
TGFβ1	ACTGGAGTTGTACGGCAGTG	GGGGCTGATCCCGTTGATT
Smad3	AGGAGAAGTGGTGCGAGAAG	CCATCCAGTGACCTGGGGAT
α-SMA	CCATGTATGTGGCTATTCAGG	AAGCGTTCGTTTCCAATGGTG
Smad7	TTCGGACAACAAGAGTCAGC	CATGGTTGCTGCATGAACTC
Beclin-1	ACCAATGTCTTCAATGCCACC	ATGGTCAAACTTGTTGTCCCAG
TNF-α	GATCGGTCCCCAAAGGGATG	CCACTTGGTGGTTTGTGAGTG
IL-6	GACAAAGCCAGAGTCCTTCAGA	TGTGACTCCAGCTTATCTCTTGG
NF-κB	CTCTGGCACAGAAGTTGGGT	TCCCGGAGTTCATCTCATAGT

Table S5. Antibody and dilution rate for Western blot/IF experiments

Antibody	Manufacturer	Cat. Number	Dilution Ratio	Experiment
α-SMA	Wanleibio	WL02510	1:500	WB
Collagen I	Wanleibio	WL0088	1:500/1:200	WB/IF
Collagen III	Wanleibio	WL03186	1:500	WB
MMP2	Wanleibio	WL03224	1:1000	WB
TIMP1	Wanleibio	WL02342	1:1000	WB
NF-κB	Wanleibio	WL01980	1:1000	WB
p-NF-κB	Wanleibio	WL02169	1:500	WB
IκBα	Wanleibio	WL01936	1:500	WB
P-IκBα	Wanleibio	WL02495	1:500	WB
IL-6	Wanleibio	WL02841	1:1000	WB
TNF-α	Bioss	bs-0078R	1:1000	WB
TGF-β1	Wanleibio	WL02998	1:1000	WB
Smad2/3	Wanleibio	WL01520	1:1000	WB
p-Smad2/3	Wanleibio	WL02305	1:500	WB
Smad3	Wanleibio	WL02288	1:300	IF
Smad7	Wanleibio	WL02975	1:500	WB
Beclin-1	Wanleibio	WL02508	1:1000	WB
ATG5	Abmart	T55766	1:1000	WB
P62	Wanleibio	WL02385	1:500	WB
LC3B	Abmart	T55992	1:500/1:50	WB/IF
GAPDH	Selleck	F0003	1:1000	WB
Peroxidase-Conjugated Goat Anti-Rabbit IgG	ZSGB Bio	ZB-2301	1:5000	WB
Alex Fluor 488 Goat Anti-Rabbit IgG	ZSGB Bio	ZF-0511	1:400	IF

Note: Western blot (WB), Immunofluorescence (IF), All IF assays listed in the table refer to cell immunofluorescence experiments.

References:

70. Chain, E.P.o.C.i.t.F. Scientific Opinion on the risks for public health related to the presence of zearalenone in food. *EFSA Journal.* **2011**, *9*, 2197.

71. Zinedine, A.; Soriano, J.M.; Moltó, J.C.; Mañes, J. Review on the toxicity, occurrence, metabolism, detoxification, regulations and intake of zearalenone: An oestrogenic mycotoxin. *Food and Chemical Toxicology.* **2007**, *45*, 1-18.

72. Gbodi, T.A.; Nwude, N.; Aliu, Y.O.; Ikediobi, C.O. The mycoflora and some mycotoxins found in maize (Zea mays) in the Plateau State of Nigeria. *Vet Hum Toxicol.* **1986**, *28*, 1-5.

73. El-Maghraby, O.M.O.; El-Kady, I.A.; Soliman, S. Mycoflora and Fusarium toxins of three types of corn grains in Egypt with special reference to production of trichothecene-toxins. *Microbiological Research.* **1995**, *150*, 225-232.

74. Wang, N.; Li, P.; Pan, J.; Wang, M.; Long, M.; Zang, J.; Yang, S. Bacillus velezensis A2 fermentation exerts a protective effect on renal injury induced by Zearalenone in mice. *Scientific Reports.* **2018**, *8*, 13646.

75. Long, M.; Yang, S.H.; Han, J.X.; Li, P.; Zhang, Y.; Dong, S.; Chen, X.; Guo, J.; Wang, J.; He, J.B. The Protective Effect of Grape-Seed Proanthocyanidin Extract on Oxidative Damage Induced by Zearalenone in Kunming Mice Liver. *Int J Mol Sci.* **2016**, *17*.

76. Long, M.; Yang, S.; Wang, Y.; Li, P.; Zhang, Y.; Dong, S.; Chen, X.; Guo, J.; He, J.; Gao, Z.; et al. The Protective Effect of Selenium on Chronic Zearalenone-Induced Reproductive System Damage in Male Mice. *Molecules.* **2016**, *21*.

77. Salah-Abbès, J.B.; Abbès, S.; Houas, Z.; Abdel-Wahhab, M.A.; Oueslati, R. Zearalenone induces immunotoxicity in mice: possible protective effects of radish extract (Raphanus sativus). *Journal of Pharmacy and Pharmacology.* **2010**, *60*, 761-770.
78. Boeira, S.P.; Funck, V.R.; Borges Filho, C.; Del'Fabbro, L.; Gomes, M.G.d.; Donato, F.; Royes, L.F.F.; Oliveira, M.S.; Jesse, C.R.; Furian, A.F. Lycopene protects against acute zearalenone-induced oxidative, endocrine, inflammatory and reproductive damages in male mice. *Chemico-Biological Interactions.* **2015**, *230*, 50-57.
79. Yang, S.; Gong, P.; Pan, J.; Wang, N.; Tong, J.; Wang, M.; Long, M.; Li, P.; He, J. Pediococcus pentosaceus xy46 Can Absorb Zearalenone and Alleviate its Toxicity to the Reproductive Systems of Male Mice. *Microorganisms.* **2019**, *7*, 266.
80. Boeira, S.P.; Filho, C.B.; Del'Fabbro, L.; Roman, S.S.; Royes, L.F.; Fighera, M.R.; Jessé, C.R.; Oliveira, M.S.; Furian, A.F. Lycopene treatment prevents hematological, reproductive and histopathological damage induced by acute zearalenone administration in male Swiss mice. *Exp Toxicol Pathol.* **2014**, *66*, 179-185.
